# Effect of amitriptyline on tetrodotoxin-resistant Na_v_1.9 currents in nociceptive trigeminal neurons

**DOI:** 10.1186/1744-8069-9-31

**Published:** 2013-06-22

**Authors:** Jingyao Liang, Xiaoyan Liu, Jianquan Zheng, Shengyuan Yu

**Affiliations:** 1 Department of Neurology, Chinese PLA General Hospital, Beijing 100853, PR China; 2 Department of Biochemical Pharmacology, Beijing Institute of Pharmacology and Toxicology, Beijing 100850, PR China

**Keywords:** Amitriptyline, Na_v_1.9, Patch clamp, Trigeminal ganglion, Pain

## Abstract

**Background:**

Amitriptyline (AMI) is tricyclic antidepressant that has been widely used to manage various chronic pains such as migraines. Its efficacy is attributed to its blockade of voltage-gated sodium channels (VGSCs). However, the effects of AMI on the tetrodotoxin-resistant (TTX-r) sodium channel Na_v_1.9 currents have been unclear to present.

**Results:**

Using a whole-cell patch clamp technique, this study showed that AMI efficiently inhibited Na_v_1.9 currents in a concentration-dependent manner and had an IC_50_ of 15.16 μM in acute isolated trigeminal ganglion (TG) neurons of the rats. 10 μM AMI significantly shifted the steady-state inactivation of Na_v_1.9 channels in the hyperpolarizing direction without affecting voltage-dependent activation. Surprisingly, neither 10 nor 50 μM AMI caused a use-dependent blockade of Na_v_1.9 currents elicited by 60 pulses at 1 Hz.

**Conclusion:**

These data suggest that AMI is a state-selective blocker of Na_v_1.9 channels in rat nociceptive trigeminal neurons, which likely contributes to the efficacy of AMI in treating various pains, including migraines.

## Background

Amitriptyline (AMI) is a tricyclic antidepressant that has also been widely used to treat different types of chronic pain, such as migraines and diabetic neuropathic pain [1,2]. The antidepressant action is known to inhibit the presynaptic reuptake of norepinephrine and/or serotonin and thus increase concentrations of these neurotransmitters at the synaptic cleft [3,4]. However, its analgesic efficacy is poorly correlated with its antidepressant action because antidepressants are analgesic in patients with chronic pain and no concomitant depression [5] and selective serotonin reuptake inhibitors (SSRIs) are typically ineffective in treating neuropathic pain [6]. Although the mechanism underlying AMI analgesic action is not fully understood, AMI inhibits voltage-gated sodium channels (VGSCs) to reduce the generation and conduction of action potentials in sensory neurons, even more than the local anesthetic bupivacaine, which could in partly explain its efficacy in relieving pain [7-9].

VGSCs Na_v_1.1-Na_v_1.9 play critical roles in electrical signaling through action potential generation and propagation in the nervous system; some specific channel subtypes have been implicated in a number of chronic pain conditions. According to their relative sensitivity to tetrodotoxin (TTX), VGSCs are classified as TTX-sensitive (TTX-s) channels (Na_v_1.1-Na_v_1.4, Na_v_1.6 and Na_v_1.7) and TTX-resistant (TTX-r) channels (Na_v_1.5, Na_v_1.8 and Na_v_1.9)[10]. Na^+^ currents blocked by AMI were first found in studies of AMI toxicity in the heart, which was supported by a study in which AMI potently inhibited recombinat cardiac hNa_v_1.5 currents [11]. AMI almost completely inhibited veratridine- or scorpion toxin-evoked efflux of endogenous dopamine (DA) and gamma-aminobutyric acid (GABA) from rat striatal slices by its blockade of Na^+^ influxes and significantly blocked Na^+^ currents in a use-dependent manner in cultured GH3 cells [12]. In bovine adrenal chromaffin cells, AMI blocked Na^+^ currents and caused a hyperpolarizing shift of the steady-state inactivation curve [13]. In cultured rat cortical neurons, AMI not only altered the activation and steady-state inactivation curves of TTX-s Na^+^ currents toward hyperpolarization but also decreased mRNA expression of Na_v_1.1, Na_v_1.2 and Na_v_1.6 channels [14]. In addition, both TTX-s and TTX-r Na^+^ currents were reduced by AMI in a dose- and holding potential-dependent manner in rat dorsal root ganglion (DRG) neurons [15]. Moreover, regardless of the heterologous expression of Na_v_1.8 in ND7/23 cells or hNa_v_1.7 in HEK293 cells, Na^+^ currents were effectively inhibited by AMI in concentration-, use- and state-dependent manners [16]. Collectively, these findings provided evidence that AMI could block a variety of VGSC currents in different manners in different cells.

To our knowledge, the effects of AMI on Na_v_1.9 currents in any cell types have not been reported, although AMI has been shown to dramatically block TTX-r Na^+^ channels in rat trigeminal ganglion (TG) neurons [17] as well as in rat DRG neurons [15]. There are at least two subtypes of TTX-r Na^+^ channels, i.e., Na_v_1.8 and Na_v_1.9, which differ in many respects, such as channel activation/inactivation kinetics and pharmacological properties. Na_v_1.8 channels are activated at relatively depolarized potentials (around −40 mV) and inactivated more slowly than TTX-s Na^+^ channels [18,19], similar to the classic TTX-r Na^+^ channels [20]. Na_v_1.9 channel activation occurs at hyperpolarized potentials (around −70 mV, close to the resting membrane potential), and its inactivation is ultraslow compared to Na_v_1.8 and TTX-s Na^+^ channels [21]. As a result, Na_v_1.9 channel activation and inactivation are widely overlapping around the resting potential, leading to the production of a persistent current [22,23]. Na_v_1.8 channels contribute to the majority of the depolarizing inward current of action potentials in neurons in which it is expressed [24,25], whereas Na_v_1.9 channels modulate resting membrane potential and responses to subthreshold stimuli and to depolarization, which could in turn amplify depolarizing inputs and increase excitability of nociceptive sensory neurons [26]. Both channels are remarkably specifically expressed in small-diameter TG and DRG neurons with thinly myelinated (Aδ) or unmyelinated axons and are likely to be implicated in the molecular mechanisms of nociception and pain [27-30].

Recently, we found that the systemic administration of AMI significantly alleviated nociceptive pains induced by electrical stimulation of the dura mater surrounding the superior sagittal sinus (SSS) in animal models of migraines [31]. Furthermore, AMI profoundly blocked Na_v_1.8 currents in concentration-, use- and state-dependent manners in acute isolated TG neurons (unpublished data). In the present study, the effects of AMI on the biophysical properties of Na_v_1.9 currents in acute isolated TG neurons were examined using whole-cell patch clamp recordings, which may provide a new molecular basis for the analgesic action of AMI.

## Results

### Recording of Na_v_1.9 currents in acute isolated TG neurons

**Figure 1:**
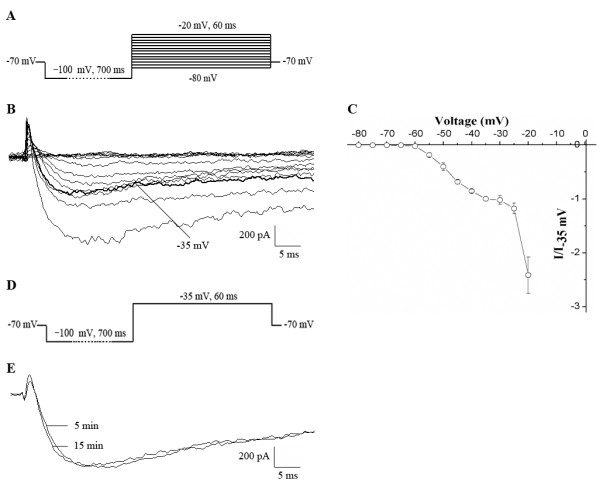
**Whole-cell patch clamp recording of TTX-r Na**_**v**_**1.9 currents in acute isolated TG neurons. A**: The protocol to elicit Na_v_1.9 currents, starting from a holding potential of −70 mV to the prepulse potential of −100 mV, and then to the voltage steps ranging from −80 to −20 mV in increments of +5 mV. **B**: Representative recordings of the Na_v_1.9 currents elicited by a series of voltage steps using the protocol in (**A**). **C**: Current/voltage (I/V) relationship of Na_v_1.9 channels with the protocol in (**A**). Each point was normalized to the amplitude of Na_v_1.9 currents at −35 mV (n = 9) **D**: The protocol of a single pulse from −100 mV to −35 mV. **E**: Peak amplitudes of Na_v_1.9 currents elicited by a single pulse at −35 mV between 5 and 15 min after whole cell activation was performed. The currents were stable during the recording time in all cells (n = 8).

In the present study, whole-cell voltage recordings were only performed on small-sized TG neurons (15–23 μm), which served as nociceptors [32]. Of 209 total neurons, 125 that showed stable recording conditions before and after compound application and washout were included for further study. According to a previous report [19,23], a voltage-clamp protocol in which neurons hyperpolarized over the course of 700 ms in response to the application of −100 mV before voltage steps applied (see protocol in Figure 1A) was used to elicit TTX-r Na_v_1.9 currents in the presence of 500 nM TTX. This step-wise protocol activated Na_v_1.9 currents first at approximately −60 mV, followed by Na_v_1.8 currents from −30 to −20 mV. Although Na_v_1.8 currents were greatest, the presence of Na_v_1.9 produced a prominent shoulder (around −35 mV) on the current/voltage (I/V) curve (Figure 1C), which before performing further experiment was examined in every TG neuron to exclude cells that do not express Na_v_1.9. Therefore, TTX-r Na^+^ currents at voltages ranging from −60 to −35 mV were mainly mediated by Na_v_1.9 channels, and the single voltage step at −35 mV was chosen to elicit the peak amplitude of Na_v_1.9 currents. The above properties are in agreement with Na_v_1.9 currents that were previously characterized in rat DRG neurons [19,23]. To observe the stabilization of its peak amplitude, Na_v_1.9 currents elicited by −35 mV were measured after whole-cell stimulation was performed. As shown in Figure 1E, the peak amplitude of Na_v_1.9 currents was relatively stable from 5 to 15 min (n = 8). All of our subsequent experiments were recorded during this time.

### Effect of different concentrations of AMI on Na_v_1.9 currents

**Figure 2:**
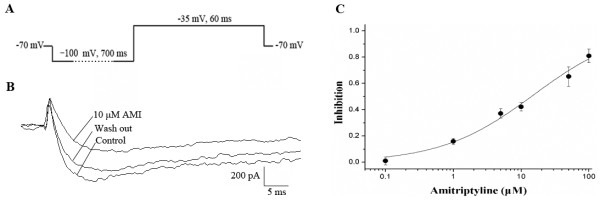
**Effects of AMI on TTX-r Na**_**v**_**1.9 currents, which were measured using whole-cell patch clamp recordings from TG neurons. A**: The protocol of a single voltage step at −35 mV. **B**: Representative recordings of the Na_v_1.9 currents elicited by a single pulse before and after perfusion with 10 μM AMI. **C**: Concentration-dependent inhibition of AMI on Nav1.9 currents with the protocol in (**A**). Each point was normalized to the control (n = 6–9).

A single voltage step protocol was used to evaluate the effect of AMI on Na_v_1.9 currents in rat TG neurons (Figure 2A). AMI caused concentration-dependent decreases in peak amplitudes of Na_v_1.9 currents, and these effects were partially reversed when AMI was washed away (Figure 2B). AMI inhibition was significant at all the following concentrations except 0.1 μM: 0.1 μM (1.01 ± 2.98%; n = 9, *P* > 0.05 ), 1 μM (15.84 ± 2.42%; n = 7, *P* < 0.05), 5 μM (37.11 ± 3.51%; n = 9, *P* < 0.05), 10 μM (42.19 ± 3.28%; n = 8, *P* < 0.05), 50 μM (65.28 ± 7.41%; n = 9, *P* < 0.05) and 100 μM (81.00 ± 5.20%; n = 6, *P* < 0.05 ) (Figure 2C). Fitting to the Hill equation revealed a half-blockade (IC_50_) at 15.16 μM with an apparent Hill coefficient of 0.64.

### Effect of AMI on Na_v_1.9 channel activation

**Figure 3:**
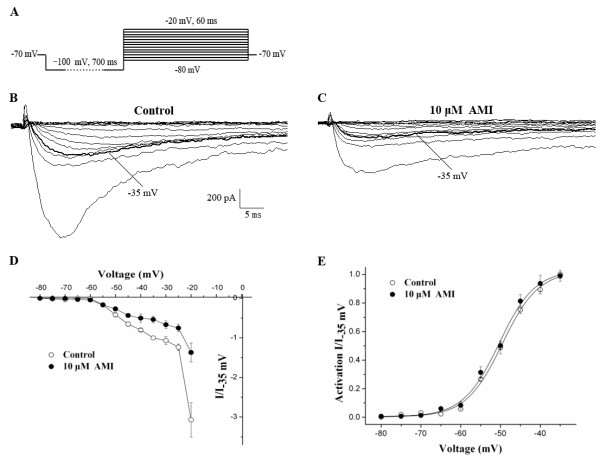
**Effect of 10 μM AMI on the voltage-activation relationship of TTX-r Na**_**v**_**1.9 currents. A**: The protocol to elicit Na_v_1.9 currents starting from a holding potential of −70 mV to the prepulse potential of −100 mV, and the voltage steps ranging from −80 to −20 mV in increments of +5 mV. **B** and **C**: Representative recordings of the Na_v_1.9 currents elicited by a series of voltage pulses using the protocol in (**A**) before and after perfusion with 10 μM AMI, respectively. **D**: The effect of 10 μM AMI on the I/V curves of Na_v_1.9 currents. Each point was normalized to the respective I_-35 mV_ currents (n = 11). **E**: Effect of 10 μM AMI on the voltage-activation relationships of Na_v_1.9 currents. Each point was normalized to its respective I_-35 mV_ of Na_v_1.9 currents (n = 11).

To examine the effects of AMI on channel activation kinetics, Na_v_1.9 currents were evoked by hyperpolarizing cells to −100 mV over the course of 700 ms, followed by the application of voltage steps ranging from −80 to −20 mV in increments of +5 mV (Figure 3A). Figures 3B and 3C show typical I/V relationships before and after perfusion with 10 μM AMI, respectively. The I/V curve was shifted upward after exposure to 10 μM AMI (Figure 3D). However, the voltage-activation curve that was fitted to the Boltzman equation only exhibited a slightly hyperpolarization after perfusion with 10 μM AMI (Figure 3E). The voltage generating half-maximal current (V_0.5act_) was −49.58 ± 0.49 mV before and −50.36 ± 0.53 mV after perfusion with 10 μM AMI (n = 11, *P* > 0.05), and the slop factor k did not change significantly (4.55 ± 0.43 before to 4.51 ± 0.47 after perfusion with 10 μM AMI, *P* > 0.05).

### Effect of AMI on Na_v_1.9 channel inactivation

**Figure 4:**
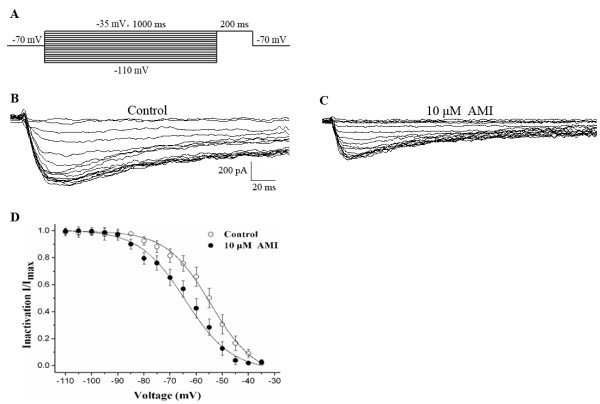
**Effect of 10 μM AMI on the steady-state inactivation relationship of TTX-r Na**_**v**_**1.9 currents. A**: The protocol to elicit steady-state inactivation of Na_v_1.9 currents starting from a holding potential of −70 mV, applying conditioning pulses ranging from −110 to −35 mV in increments of +5 mV, and applying a test pulse at −35 mV. **B** and **C**: Representative recordings of the Na_v_1.9 currents elicited by a series of test pulses using the protocol in (**A**) before and after exposure to 10 μM AMI, respectively. **D**: The effect of 10 μM AMI on the steady-state inactivation relationship of Na_v_1.9 currents. Each point was normalized to its respective maximal Na_v_1.9 currents (n = 11).

To measure the steady-state inactivation of Na_v_1.9 channels, double-pulse protocols starting from a holding potential of −70 mV were used. Conditioning pulses from −110 mV to −35 mV were performed over 1 s to ensure that Na_v_1.9 channels were entirely inactivated (Figure 4A). Figures 4B and C show the recorded channel responses to test pulses (the voltage step at −35 mV for 200 ms) before and after exposure to 10 μM AMI, respectively. The steady-state inactivation, fit to the Boltzmann equation exhibited a significant hyperpolarization (Figure 4D). Half-maximal steady-state inactivation (V_0.5inact_) was −54.50 ± 0.77 mV before and −64.17 ± 1.09 mV after perfusion with 10 μM AMI (n = 11, *P* < 0.05), and there was no statistical change in the slop factor k (8.47 ± 0.56 before to 8.86 ± 0.92 after perfusion with 10 μM AMI, *P* > 0.05).

### Effect of AMI on use-dependent blockade of Na_v_1.9 channels

**Figure 5:**
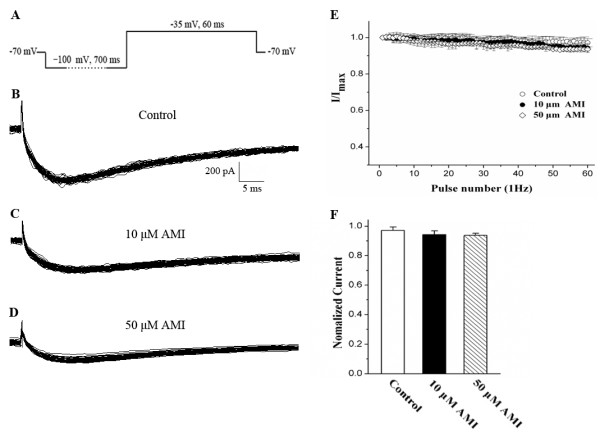
**Effect of 10 and 50 μM AMI on the use-dependent relationship of TTX-r Na**_**v**_**1.9 currents. A**: The protocol of a single voltage step at −35 mV. **B**, **C** and **D**: Representative recordings of the Na_v_1.9 currents elicited by 60 pulses at 1 Hz before and after perfusion with 10 and 50 μM AMI, respectively. **E**: The effect of 10 and 50 μM AMI on the use-dependent relationship of Na_v_1.9 currents. Each point was normalized to the respective first Na_v_1.9 current (n = 9). **F**: The current at the 60th pulse normalized to the current of the first pulse, was not significantly decreased after perfusion with 10 or 50 μM AMI compared to that of the controls (n = 9, *P* > 0.05).

To study whether its channels could be blocked by AMI in use-dependent manner, Na_v_1.9 channels were activated at 1 Hz by 60 test pulses at −35 mV from a hyperpolarized potential of −100 mV (Figure 5A). Treatment with 10 μM AMI significantly reduced the peak amplitude of Na_v_1.9 currents compared to those in the absence of AMI (Figure 5B); however, AMI had little effect on the use-dependence of Na_v_1.9 channels (Figure 5C). The amplitude of the 60th Na_v_1.9 current only slightly decreased to 94.29 ± 2.50% of the first current during the 10 μM AMI perfusion, and no difference was observed when compared to control currents (97.13 ± 2.26%, n = 9, *P* > 0.05; Figure 5E). To rule out the influence of the concentration on use-dependent blockade, 50 μM AMI was used in a subsequent test. Similar to the 10 μM experiments, 50 μM AMI had little effect on the use-dependence of Na_v_1.9 channels (93.65 ± 1.43% of the first one, n = 9; Figures 5D and 5E). These results indicated that AMI did not significantly contribute to the use-dependent blockade of Na_v_1.9 currents when stimulated by 60 pulses at 1 Hz.

## Discussion

Although AMI has been widely reported to block some subtypes of VGSCs, including Na_v_1.5, Na_v_1.7 and Na_v_1.8 [11,16], to our knowledge, this is the first report that studies the effects of AMI on TTX-r Na_v_1.9 channels in TG neurons.

The present results showed that AMI efficiently inhibited Na_v_1.9 channels in rat TG neurons in a concentration-dependent manner and had an IC_50_ of 15.16 μM, consistent with previous findings that AMI blocked TTX-r Na^+^ currents in rat TG neurons (IC_50_ of AMI was 15.8 μM) [17]. In patients with depression or neuropathic pain who receive daily doses of 10 to 300 mg AMI, plasma steady-state concentrations range from 0.36 to 0.90 μM [33]. The concentrations of AMI used in this study are higher than the clinically relevant plasma concentrations. However, even at clinically relevant concentrations between 0.1 and 1 μM, the peak Na^+^ currents were still decreased by approximately 1–15%. However, the brain and plasma concentration ratios of AMI observed in chronically treated rats were found to be more than 20:1, which was similar to levels reported in humans [34,35]. In this case, the IC_50_ value for Na_v_1.9 channels would be similar to the concentration of AMI found in the brain: it is thus possible that Na_v_1.9 channels are effectively inhibited in TG neurons. Unlike drugs that block both TTX-s and TTX-r Na^+^ channels in rat DRG neurons by modulating Na^+^ channel activation and inactivation kinetics [15], AMI produced only a prominent hyperpolarizing shift in the steady-state inactivation curves of Na_v_1.9 channels and had no significant effects on the channel activation kinetics in rat TG neurons, indicating that the binding of AMI to Na_v_1.9 channels was state-dependent. This phenomenon was similar to previous reports on the inhibition of TTX-r Na^+^ channels in rat TG neurons [17]. The discrepancy in these findings may be due to differences in tissue sources and the experimental protocols.

Previous studies have shown that the blockade of TTX-s and TTX-r Na^+^ channels and Na_v_1.8 channels by local anesthetics and AMI is highly use-dependent [16,36,37]. However, there was no use-dependent blockade in the presence of 10 or 50 μM AMI at 1 Hz stimulation in this study. This use-dependent blockade would result from the binding of an antagonist ligand to inactivated channels that are more prevalent during repetitive stimulation and from the dissociation of the antagonist from the inactivated states with a time constant slower than the frequency of the pulses, which means that use-dependent blockade arises from the slow recovery of antagonist-bound channels due to an interaction between antagonist and inactivated states [16,37]. According to this hypothesis, the dissociation time constant of AMI from inactivated Na_v_1.9 channels might be faster than the frequency of the pulses (1 Hz) used in this study. Clearly, only one frequency was tested in this experiment; higher frequencies, such as 5, 10 or 20 Hz, were not investigated due to limitations in the protocol, in which the spent time of a single voltage step was approximately 900 ms (Figure 5A). These inconsistent findings might also be explained by the presence of the different binding sites. For example, local anesthetics and AMI are known to block Na_v_1.8 channels in a use-dependent manner by binding to the same binding site, which are located within the ion-conducting pore [8,38,39]. Selective Na_v_1.8 channel blockers A-803467 and A-887826 do not cause use-dependent blockage and were instead thought to recognize a binding site that is distinct from the binding sites for use-dependent blockers [40]. It was recently proposed that Na_v_1.9 currents exhibit ultraslow activation and inactivation kinetics, which is likely the product of a substantially different amino acid sequence, especially in the voltage-sensing regions, compared to other Na^+^ channel subtypes [22,41]. Therefore, further studies on the dissociation time constant of AMI from the inactivated states of Na_v_1.9 channels will help address this issue.

In addition to having the effect on Na_v_1.9 currents in the present study, AMI was also reported to inhibit Na_v_1.8 channels heterologously expressed in ND7/23 cells in concentration- and use-dependent manners, and to change activation and inactivation kinetics of Na_v_1.8 channels [16]. Similar results were also obtained from our study on modulation of Na_v_1.8 channels by AMI in TG neurons (unpublished data). Na_v_1.8 and Na_v_1.9 channels may be individually expressed or co-expressed in the small diameter TG neurons, so the potential contamination with Na_v_1.8 current and possible impact on AMI behavior may be included in the present study, although Na_v_1.8 and Na_v_1.9 currents can be distinguished by the experimental protocols [19,23] and the I/V curve which was examined in every TG neuron to validate presence of Na_v_1.9. This study showed that AMI had no effects on the activation kinetics and had no use-dependent blockade of Na_v_1.9. However, it is well documented that AMI significantly changed the activation kinetics and caused use-dependent blockade of Na_v_1.8 [16]. These results suggested that even if it had, it would only a little the potential contamination with Na_v_1.8 current and a little impact on AMI behavior in the present study.

Persistent subthreshold Na^+^ currents, carried primarily by Na_v_1.9 channels that are expressed exclusively in small nociceptive TG and DRG neurons [27-30], are known to lessen spike threshold and eventually facilitate maintained spiking [23,42]. The loss of Na_v_1.9-mediated Na^+^ currents was associated with the inability of neurons to generate a large variety of electrophysiological behaviors, including subthreshold regenerative depolarization, active hyperpolarizing responses, oscillatory bursting discharges, plateau potentials and bistable membranes [43]. In Na_v_1.9 knock-out mice, there is a loss of persistent currents and blunted or missing pain behaviors induced by complete Freund adjuvant (CFA), carrageenan, formalin, and prostaglandin E2 (PGE2) [44] or in response to inflammatory mediators, such as bradykinin, serotonin, interleukin-1beta, and P2X3 and P2Y receptor agonists [45]. Consistently, antisense-based Na_v_1.9 gene silencing in rats attenuated carrageenan-induced heat and mechanical pain allodynia [46]. Response to pain is relevant to the persistent currents, as demonstrated by electrophysiological studies in isolated primary sensory neurons, where inflammatory mediators such as PGE2 and serotonin [44,47], as well as some secreted proteins (e.g., glial-derived neurotrophic factor (GDNF) [48]), or activators of G protein pathways [26], have been reported to increase Na_v_1.9 currents.

Migraines are the most common headache disorder and affect more than 10% of the general population [49,50]. Migraines are thought to arise from the activation and sensitization of the trigeminovascular system, followed by the release of inflammatory mediators from the trigeminal system, with a consequent vasodilation of innervate intracranial blood vessels and generation of neurogenic inflammation [51]. Such inflammation causes hyperexcitability of TG neurons (peripheral sensitization) and the second-order sensory neurons (central sensitization) [52,53]. The above-mentioned PGE2, interleukin-1 beta, and G protein-coupled P2X3 and P2Y receptors, which are known to functionally regulate Na_v_1.9 channels during inflammation, have also been closely linked to the pathophysiology of migraines in a variety of experimental and clinical studies [54-57]. In addition, immunohistochemical experiments had shown that P2X3, bradykinin B2, and transient receptor potential vanilloid 1 (TRPV1) receptors are highly co-localized with Na_v_1.9 channels in nociceptor sensory neurons [45]. TRPV1 receptors have been implicated as new therapeutic targets for the treatment of migraines [58]. Although there is not a direct link between migraines and Na_v_1.9 channels, AMI has been widely used for the prophylactic treatment of migraines and has demonstrated clear success in clinical practice. These results were supported by our findings that AMI efficiently blocked Na_v_1.9 currents, which might help, at least in part, understand the mechanism underlying AMI efficacy in migraine pain.

## Conclusion

In summary, the present results demonstrate that AMI is a state-selective blocker of Na_v_1.9 channels in nociceptive trigeminal neurons, which likely contributes to the analgesic action of AMI in various pains including migraines.

## Methods

### Preparation of TG neurons

All experimental procedures were approved by the Committee of Animal Use for Research and Education of the Laboratory Animals Center of the Chinese PLA General Hospital (Beijing, PR China) and were consistent with the ethical guidelines recommended by the International Association for the Study of Pain in conscious animals [59]. Efforts were made to minimize the animals’ suffering. TG neurons from 7-day-old neonatal Sprague–Dawley rats (The Academy of Military Medical Sciences, Beijing, PR China) were prepared using a modified version of a previously described method [60]. Briefly, rats were deeply anesthetized by intraperitoneal injection of euthasol (0.1 mg/kg) and decapitated. A pair of the TGs were rapidly dissected from each animal, washed several times in ice-cold Hank’s Balanced Salt Solution (HBSS; Life Technology, MD), and then dissociated by mechanical disruption and incubated in 2 mL HBSS containing 0.25% trypsin at 37°C for 25 min. The tissues were washed twice in DMEM (high glucose) (Hyclone, Logan, UT) and resuspended in DMEM with 10% fetal bovine serum, 10% heat-inactivated horse serum and 1% L-glutamine, and triturated with a flame-polished Pasteur pipette to dissociate individual cells. Subsequently, cells were plated onto poly-L-lysine-coated glass coverslips (12 mm diameter) placed in 24-well plates, and then maintained in a humidified atmosphere of 95% air and 5% CO_2_ at 37°C. The cells were used for recordings between 2 and 10 h after plating.

### Patch clamp recordings

The whole-cell patch clamp recordings were performed at room temperature; currents were measured with an Axopatch-200B (Axon Instruments, Inc., Foster City, CA, USA) and recorded with pClamp 8.2 software (Axon Instruments, Inc., Foster City, CA, USA). The output was digitized with a Digidata 1322A converter (Axon Instruments, Inc., Foster City, CA, USA). Patch pipettes were made by a two-step vertical puller (Narishige Scientific Instrument Laboratory, Tokyo, Japan; model PP-83) from borosilicate glass and had resistances between 2 to 3 MΩ after perfusion of internal solution through the pipette. Cells in the glass coverslip dishes were placed in a recording chamber and visualized with the phase contrast microscopy on an inverted microscope (Nikon, Tokyo, Japan). Currents were recorded from small TG neurons (15–23 μm diameter). Experiments were performed at a holding potential of −70 mV for Na_v_1.9 currents. After gigaohm seal formation and membrane disruption, the whole cell capacitance was cancelled and series resistance was compensated for (> 80%). Data were low-pass-filtered at 2 kHz, sampled at 10 kHz, and acquired with the pulse protocol. The liquid junction potential between internal and external solutions was −5 mV on average and was used to correct for the recorded membrane potential. The pipette solution was composed of the following (in mM): 140 CsCl, 10 NaCl, 1 MgCl•6H_2_O, 0.5 CaCl_2_, 5 EGTA, 10 HEPES and 2 Na_2_-ATP, and adjusted to pH 7.4 with CsOH (320 mosm). Extracellular solution contained the following (in mM): 120 NaCl, 5 KCl, 30 TEA-Cl, 10 Glucose, 10 HEPES, 10 4-AP, 2 CaCl_2_, 0.1 CdCl_2_, 2 MgCl•6H_2_O and 0.0005 TTX, and adjusted to pH 7.4 with NaOH (310 mosm). The TEA-Cl, CdCl_2_ and TTX were used to inhibit endogenous K^+^, Ca^2+^ and TTX-s sodium currents, respectively.

### Drugs and chemicals used

AMI, TTX, trypsin, L-glutamine, poly-L-lysine, HEPES, EGTA, TEA-Cl, Na_2_-ATP, CdCl_2_, CsOH and CsCl were purchased from Sigma. Other chemical reagents used were of analytic grade. AMI was prepared as a 100 mM stock solution in distilled water and further dilutions were made fresh in extracellular solution on the day of each experiment. AMI was continuously administered (approximately 1 mL/min) to the cells via superfusion polyethylene tubes during the recording procedure.

### Data analysis

Data were analyzed using pCLAMP 10.0 (Axon instruments, USA) and Origin 7.5 (Microcal Software, Northampton, MA, USA) software. Concentration-response curves were fit to the Hill function: *I*_drug_*/I*_control_ = 1/[1 + (C/IC_50_)^H^], where *I*_drug_*/I*_control_ is fractional blockade, C is the drug concentration, IC_50_ is the drug concentration that causes 50% blockade, and H is the Hill coefficient. The voltage-activation curves and the steady-state inactivation curves were fit with the Boltzmann function: *I/I*_max_ = 1 − 1/(1 + exp[(V_m_ − V_0.5 act_)/k)] and *I/I*_max_ = 1/(1 + exp[(V_m_ − V_0.5 inact_)/k)], respectively, where Imax is maximal current, V_m_ is the prepulse voltage, V_0.5_ is the voltage generating half maximal current, and k is the slope factor of the curves. All data are presented as the mean ± SEM. Statistical significance was assessed using SPSS 13.0 (SPSS Inc, Chicago, IL). Student’s t-test analysis was used to assess differences between means from two groups. One-way ANOVA of variance followed by Dunnett post-testing was performed to assess differences than two more groups. A *P* value < 0.05 was considered to be significant.

## Abbreviations

AMI: Amitriptyline; CFA: Complete Freund adjuvant; DA: Dopamine; DRG: Dorsal root ganglion; GABA: Gamma-aminobutyric acid; GDNF: Glial-derived neurotrophic factor; PGE2: Prostaglandin E2; SSRIs: Selective serotonin reuptake inhibitors; SSS: Superior sagittal sinus; TG: Trigeminal ganglion; TRPV1: Transient receptor potential vanilloid 1; TTX: Tetrodotoxin; TTX-r: Tetrodotoxin-resistant; TTX-s: TTX-sensitive; VGSCs: Voltage-gated sodium channels.

## Competing interests

The authors declare that they have no competing interests.

## Authors’ contributions

JYL and XYL performed the patch clamp recordings in TG neurons. JQZ was partially involved in experimental design and guiding. SYY is the corresponding author. All authors read and approved the final manuscript.
